# Percutaneous Radiofrequency Ablation for Painful Spinal Metastases Resulting in Resolution of Epidural Disease: A Case Report

**DOI:** 10.7759/cureus.2579

**Published:** 2018-05-04

**Authors:** John A Holbert, Dan T Nguyen

**Affiliations:** 1 College of Medicine, Penn State University College of Medicine; 2 Interventional Neuroradiology, Penn State Hershey Medical Center, Hershey, USA

**Keywords:** bone metastases, interventional radiology, spinal metastases, back pain, radiofrequency ablation

## Abstract

Percutaneous image-guided ablation is used for treatment of both benign and malignant osseous lesions often leading to substantial pain relief and local tumor control. Paired with vertebral augmentation of the affected vertebra, patients can often become functional and experience significant pain reduction. However, bone ablation must be paired with various modalities of treatment as it only provides pain relief and local tumor control and does not address systemic metastatic disease.

We describe a case of metastatic prostate cancer with epidural extension treated with percutaneous image-guided radiofrequency ablation and vertebral augmentation leading to substantial pain relief as well as resolution of the epidural disease as evidenced by short-term follow-up magnetic resonance imaging (MRI). To the best of our knowledge, the resolution of epidural disease has not been described before. This case highlights the potential of ablative therapy in metastatic bone disease, particularly in the presence of epidural disease.

## Introduction

Percutaneous image-guided ablation has become a common and effective management option for patients with benign and malignant osseous lesions. Multiple types of ablative modalities are available including radiofrequency, cryoablation, high intensity focused ultrasound, laser, and microwave techniques. Multiple studies have specifically centered around radiofrequency ablation for osseous lesions. Goetz et al. published the first case series in 2004 showing its palliative efficacy [1]. The safety and efficacy of radiofrequency ablation with vertebral augmentation is a safe and effective way to treat painful vertebral metastases [2-4]. The success of this technique to provide both pain palliation and the means to control local tumor spread has been well documented. However, extension of the metastasis to the epidural space is often an excluding factor for this treatment. This case describes the successful treatment of painful metastatic prostate cancer to the thoracic and lumbar spine and more importantly, the significant resolution of the epidural disease.

## Case presentation

An 81-year-old male presented for consideration of vertebral augmentation due to diagnosis of stage IV, metastatic prostate adenocarcinoma, and worsening back pain. Lupron therapy was initiated at diagnosis four months prior. Docetaxel treatment was planned for six cycles but was subsequently stopped after the first cycle secondary to side effects. No radiation therapy was previously given. PSA level was 120.73 at diagnosis and 0.6 before radiofrequency ablation.

**Figure 1:**
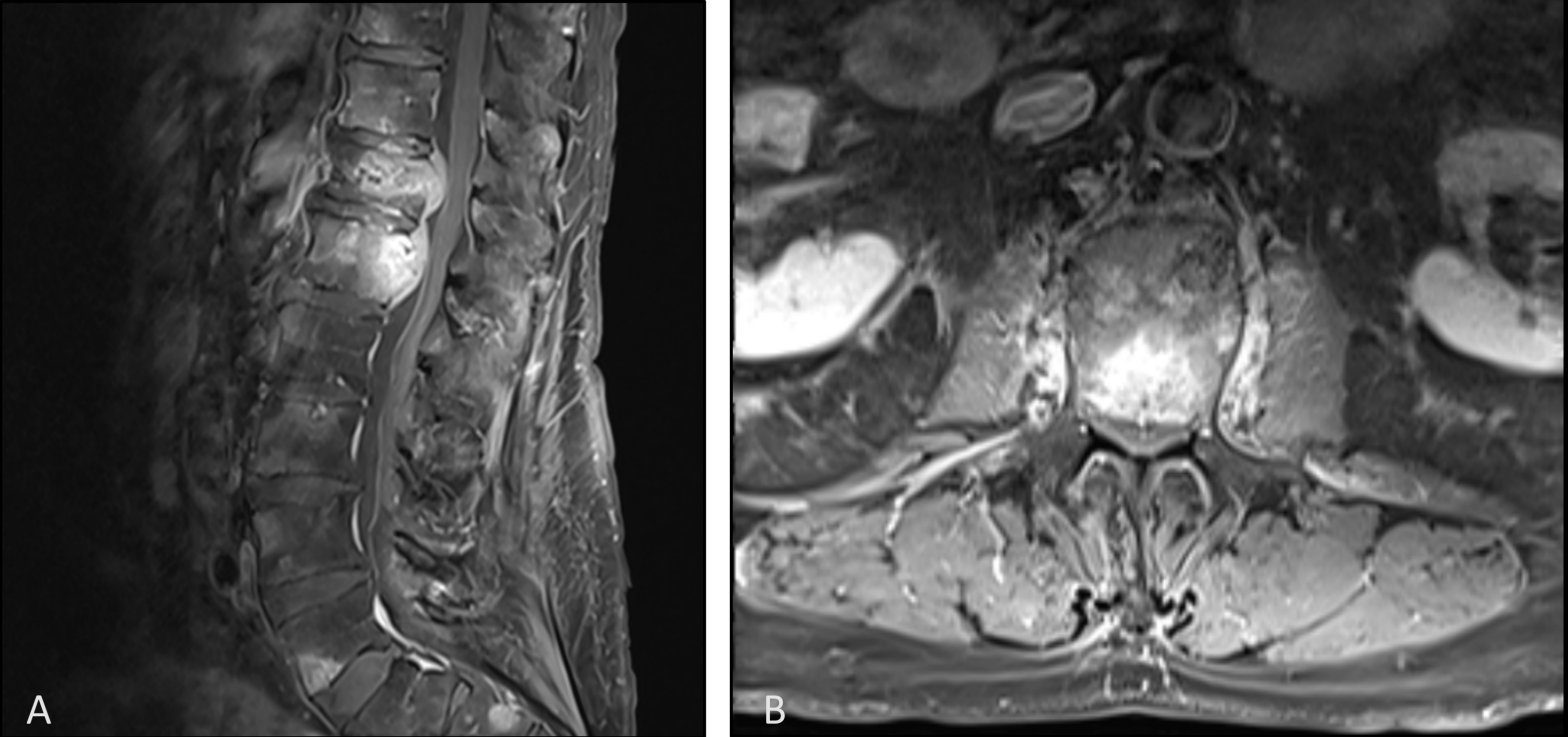
Pre-treatment contrast enhanced T1-weighted magnetic resonance imaging of the sagittal spine (A) and axial spine at L1 (B) showing vertebral metastases and epidural involvement

At the first appointment, the patient reported mild back pain and required a walker but was able to ambulate without difficulty. He did have pain upon palpation of the thoracolumbar junctional level. He did not have any neurologic deficit at presentation. Computed tomography (CT) scans showed 40% compression deformity of T12. Magnetic resonance imaging (MRI) showed pathologic involvement of T12 and L1 and metastatic involvement of the epidural component, resulting in 40% spinal canal stenosis (Figure 1). At this time, vertebral augmentation was recommended although it was believed the epidural component would not be addressed and Radiation Oncology would need to be consulted. In a short period of two months, the patient’s condition deteriorated where he was wheelchair bound due to severe pain, not controlled with NSAIDS or opioids. In addition, repeat studies showed further tumor infiltration involving T11, prompting augmentation of T11, in addition to T12 and L1.

**Figure 2:**
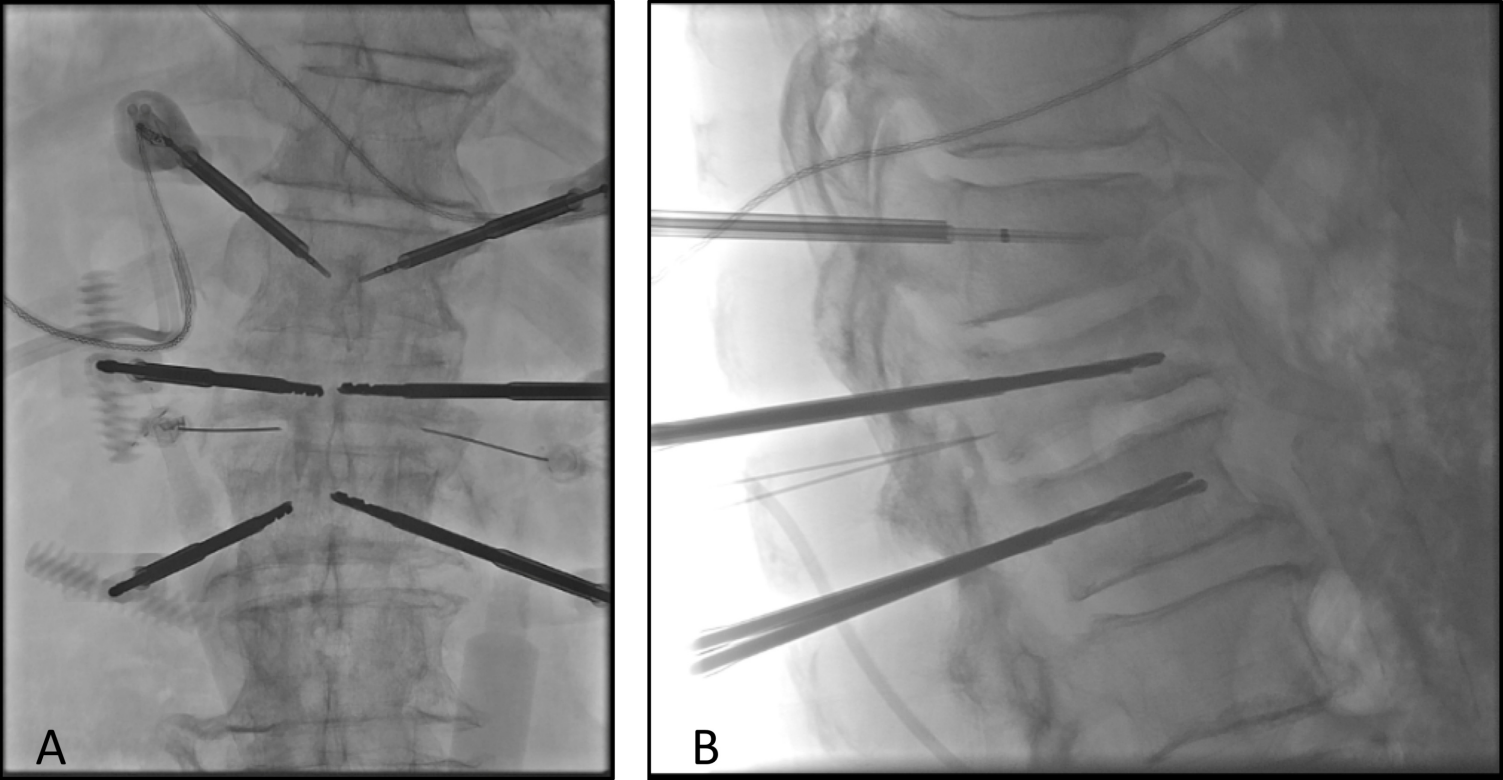
Intraoperative AP (A) and lateral (B) radiographic views

**Figure 3:**
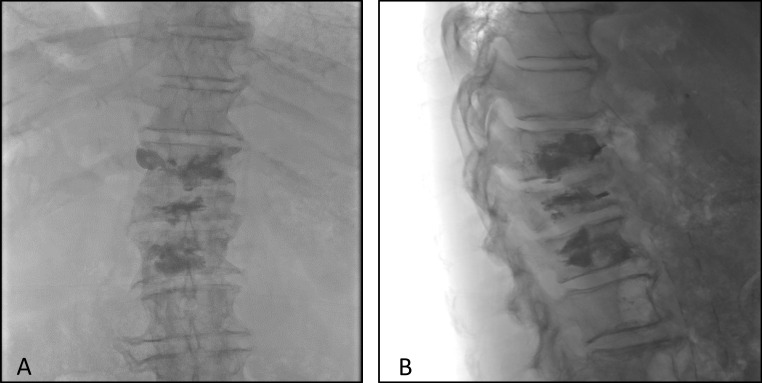
AP (A) and lateral (B) radiographic views post radiofrequency ablation and vertebral augmentation

The procedure was performed under monitored anesthesia care (MAC) and fluoroscopic guided imaging. Under this image guidance, 10-gauge introducer needles were advanced into the T11, T12, and L1 vertebral levels using a bilateral transpedicular approach (Figure 2). A drill and osteotome were used to create cavities at the anterior aspect of the vertebral bodies. Bilateral 17-gauge bipolar radiofrequency probes were advanced into the vertebral cavities and simultaneous application of radiofrequency energy was performed as part of the protocol for volumetric ablation of the vertebral bodies. These were done in serial at T11, T12, and L1 vertebral bodies for approximately 15 minutes for each level. Lastly, methylmethacrylate was injected into the vertebral bodies of T11, T12, and L1 (Figure 3) for vertebral stability. No complications occurred during the surgery and the patient was discharged the same day.

**Figure 4:**
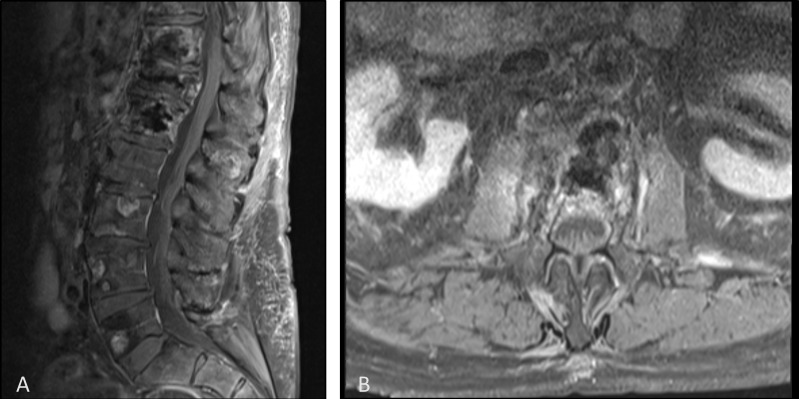
Post-treatment contrast enhanced T1 weighted magnetic resonance imaging of the sagittal spine (A) and axial spine at L1 (B) showing vertebral augmentation changes and resolution of epidural disease and spinal compression

The patient reported no pain at the three-week follow-up and he was able to ambulate without assistance and continued to increase daily activities. He also no longer required any pain medication. He continued to be pain-free at the eight-week follow-up and repeat MRI showed stable vertebral changes and complete resolution of epidural disease at the T12 and L1 level (Figure 4). At nine-months post-op, the patient still had no pain and returned back to his normal activities.

## Discussion

Spinal metastases occur in a significant portion of cancer patients, estimated at 40%. As life expectancy increases, so will the number of these cases [5]. Spinal metastases often lead to severe pain and decline in quality of life that cannot be managed through pharmacological options. The lytic component of metastatic prostate cancer creates a local bone weakening effect. This contributes to both the painful aspect of the disease and increased risk for vertebral collapse. Image-guided radiofrequency ablation has great potential for providing significant pain relief for patients with metastatic disease of the spine as shown in this case. Multiple studies have demonstrated the efficacy of this treatment for improving the quality of life for these cancer patients [5].

Radiofrequency ablation leads to tissue breakdown through frictional heat created by high-frequency alternating current [6]. The zone of ablation created by the probe allows for controlled treatment of the target lesion. While ablation should not be used as the single treatment of metastatic cancer, it does have potential to control local tumor spread and may even lead to regression of surrounding tumor. One study showed 89% and 70% of local control at three months and one year post procedure respectively [7]. Ablation does not address the mechanical instability associated with these lesions, therefore, when paired with vertebral augmentation, the affected vertebra is stabilized to prevent future spinal complications.

One complicating factor in the treatment of spinal metastases is the involvement of epidural tissue. Epidural disease is not expected to be addressed by radiofrequency ablation and is often considered a contraindication. In this case, after follow-up MRI it was clear that the epidural disease resolved secondary to the radiofrequency ablation of the vertebrae. This case highlights that the presence of epidural spread should not be an absolute contraindication for therapy and in fact, may lead to regression of the epidural disease. We believe that epidural disease in the setting of radiofrequency ablation and vertebral augmentation should be further explored.

## Conclusions

Spinal metastases are a serious complication of many cancers and lead to debilitating pain and decline in quality of life. Radiofrequency ablation and vertebral augmentation is a promising treatment for patients that can lead to significant improvement in quality of life. The involvement of epidural disease in the setting of radiofrequency ablation has not been well documented. This case shows that epidural disease should not be an absolute contraindication for treatment and may even be addressed by radiofrequency ablation and vertebral augmentation.
